# Removal of Protein-Bound Uremic Toxins by Mixed Matrix Membranes of Cellulose Acetate/Silica/MOF

**DOI:** 10.3390/membranes16070232

**Published:** 2026-07-02

**Authors:** João M. Santos Dionísio, Miguel P. da Silva, Ricardo F. S. Pereira, Tânia Frade, Tiago J. Ferreira, Moisés Luzia Pinto, Maria Norberta de Pinho

**Affiliations:** 1 Center of Physics and Engineering of Advanced Materials (CeFEMA), Instituto Superior Técnico, Universidade de Lisboa, Av. Rovisco Pais, n. 1, 1049-001 Lisboa, Portugal; 2 Department of Chemical Engineering, Instituto Superior Técnico (IST), Universidade de Lisboa, Av. Rovisco Pais 1, 1049-001 Lisboa, Portugal; 3 iBB-Institute for Bioengineering and Biosciences, Department of Bioengineering, Instituto Superior Técnico, Universidade de Lisboa, Av. Rovisco Pais, 1049-001 Lisboa, Portugal; 4 Associate Laboratory i4HB-Institute for Health and Bioeconomy, Instituto Superior Técnico, Universidade de Lisboa, Av. Rovisco Pais, 1049-001 Lisboa, Portugal; 5 Centro de Recursos Naturais e Ambiente, Department of Chemical Engineering, Instituto Superior Técnico (IST), Universidade de Lisboa, Av. Rovisco Pais, n. 1, 1049-001 Lisboa, Portugal

**Keywords:** hemodialysis, mixed matrix membranes, protein-bound uremic toxins, metal–organic frameworks (MOFs), p-cresyl sulfate

## Abstract

Adsorption therapies in hemodialysis have emerged as an innovative approach for removing protein-bound uremic toxins (PBUTs). The present work focuses on the enhancement of the adsorption capacity of hemodialysis membranes through the incorporation of Metal–Organic Frameworks (MOFs). The removal capacity of PBUT p-cresyl sulfate by cellulose acetate (CA)/silica (SiO_2_)/MOF mixed matrix membranes was investigated with two types of MOFs, UiO-66 which synthesis and characterization has been previously reported, and UiO-66-NH_2_. The UiO-66-NH_2_ MOFs were synthesized and characterized by infrared spectroscopy, X-ray diffraction, nitrogen adsorption–desorption equilibrium at −196 °C, and thermogravimetry analysis. Both mixed matrix membranes were synthesized by coupling the phase inversion technique with the sol–gel method and with casting solutions incorporating the MOF dispersions. The two membrane types of MOFs were characterized in terms of hydraulic permeability, molecular weight cut-off, and rejection coefficients to pCS and bovine serum albumin (BSA). The mixed matrix membranes CA/SiO_2_/UiO-66-NH_2_ exhibited lower permeability and molecular weight cut-off when compared to the CA/SiO_2_/UiO-66 ones. In permeation tests simulating a hemodialysis session with a feed solution of 100 ppm pCS and 35 g/L BSA, it is shown the improved performance of MOFs membranes as the rejection coefficients of free pCS is 0.2% for the CA22/SiO_2_/UiO-66 membrane with 1.5% of MOF and 2.6% for the CA22/SiO_2_/UiO-66-NH_2_ membrane with 2% of MOF. The capacity of these MOF membranes in removing pCS bound to BSA was addressed through the development of a new methodology to quantify the pCS free and bound to BSA. The CA22/SiO_2_/UiO-66 membrane with 1.5% of MOF has a removal capacity of 99.8% and the CA22/SiO_2_/UiO-66-NH_2_ membrane with 2% of MOF 95.9%. Based on these results, it is concluded that the mixed matrix membranes CA22/SiO_2_/UiO-66 and CA22/SiO_2_/UiO-66-NH_2_ are promising candidates for PBUTs removal in hemodialysis.

## 1. Introduction

Approximately three million patients with end-stage renal disease receive renal replacement therapies (RRT) such as hemodialysis, which only partially restore kidney function, as they are only effective at partially removing small solutes that are free in the blood, leaving larger uremic toxins and those bound to plasma proteins, PBUTs, circulating in the bloodstream [1,2,3].

In the 1990s, the development of dialysis equipment and membranes capable of combining mass transfer mechanisms through both diffusion and convection paved the way for new RRT such as high-flux hemodialysis and hemodiafiltration [4]. Numerous materials are used for hemodialysis membranes, including cellulose-based materials such as regenerated cellulose and cellulose acetate, as well as synthetic polymers like polyacrylonitrile, ethylene vinyl alcohol, polysulfone, and polyethersulfone [5]. The membranes have ultrafiltration (UF) sieving characteristics that assure the retention of vital compounds of the blood like the plasma proteins and the removal of the toxins with molecular weight below the membrane molecular weight cut-off (MWCO).

Since albumin is the most abundant plasma protein, it plays a crucial role in binding various compounds to its structure, including uremic toxins and medications, thanks to its two binding sites for toxins: Sudlow I and Sudlow II. Bovine serum albumin (BSA) has been widely used as a model protein in experimental work due to its structural homology with human serum albumin (HSA), easiness of preparation, high purity and good water solubility [6,7].

The accumulation of PBUTs in patients with chronic kidney disease (CKD) can lead to various systemic effects. These toxins are known for their harmful action on numerous tissues, having a significant impact on the cardiovascular system. High levels of indoxyl sulfate (IS) and p-cresyl sulfate (pCS) in plasma have been indicators of cardiovascular events and vascular diseases, including atherosclerosis, endothelial injury, and vascular calcification [6,8].

Adsorption therapies represent an innovative strategy for the removal of uremic toxins in patients with CKD. Despite their considerable effectiveness, some challenges remain, such as the associated costs and biocompatibility. These therapies rely on the ability of certain adsorbent materials to selectively capture PBUTs from the blood, resulting in a decrease in the fraction bound to proteins [9].

The high adsorption capacity of activated carbon and other adsorbent materials has led to a significant reduction in toxin concentrations [6,9,10]. More recently, metal–organic frameworks (MOFs) have been investigated as potential adsorbents for PBUTs [7,11,12,13]. In the research conducted by Kato et al. [14], zirconium-based MOFs, NU-1000 and UiO-66, were studied for the adsorption of pCS, indoxyl sulfate, and hippuric acid, revealing some promising results. Dymek et al. [15] found that maximum adsorption of hippuric acid and 3-indoleacetic acid was achieved with the MOF UiO-66-NH_2_. Regarding in vitro cytotoxicity tests and hemolytic activity assays, these studies indicated that a UiO-66-based material could be considered potentially safe for hemodialysis processes in living organisms [16].

The present work addresses the capacity of ultrafiltration with mixed matrix membranes of cellulose acetate/silica/MOF to remove pCS bound to BSA due to the membranes adsorptive character as the result of the incorporation of the zirconium-based MOFs, UiO-66 and its amino-functionalized derivative UiO-66-NH_2_.

The UF is carried out with solutions of pCS and BSA where the content of pCS is 100 ppm, the typical value for a patient with CKD. The fractions of pCS free and bound to BSA are quantified in the feed and in the retentate UF streams.

## 2. Materials and Methods

### 2.1. Analytical Methods

In the present work, a Shimadzu UV-Vis spectrophotometer (UV-1700 PharmaSpec, Kyoto, Japan) and a Thermo Scientific microplate reader (Multiskan GO, Waltham, MA, USA) were used, with the latter being used to obtain the absorbance spectra, λ: 200–800 nm, of the analyzed samples of pCS (synthetized as described in [17]), BSA (Panreac AppliChem, Darmstadt, Germany), and pCS bound to BSA. The TOC analyzer used is a combustion-based analyzer (SHIMADZU, TOC-VCSH, Kyoto, Japan) that quantified the carbon content in each sample.

### 2.2. UiO-66 and CA22/SiO_2_/UiO-66 Membranes Synthesis

The synthesis of UiO-66 and of the CA22/SiO_2_/UiO-66 mixed matrix membranes incorporating 1%, 1.5%, 2% and 2.5% of UiO-66 is described in [1].

### 2.3. UiO-66-NH_2_ Synthesis and Characterization

First, 14.8400 g (81.9 mmol) of 2-aminoterephthalic acid (H_2_BDC-NH_2_, 181.15 g/mol, 99%, Thermo Scientific) and 26.3050 g (81.6 mmol) of zirconyl chloride octahydrate (ZrOCl_2_·8H2O, 98%, Alfa Aesar, Haverhill, MA, USA) were weighed in separate beakers. The reagents were dissolved in 35 and 220 mL of dimethylformamide (DMF, HCON(CH_3_)_2_, 73.09 g/mol, ≥99.9%, Carlo Erba, Milan, Italy), respectively. The content of each beaker was added to a Lab1st reactor already containing 250 mL of DMF with stirring at 80 rpm. The zirconyl chloride octahydrate was added first. After this, 27 mL of hydrochloric acid (HCl, 1M, Thermo Scientific) was added dropwise to the reactor. The stirring was changed to 170 rpm and the reaction took place under reflux at 110 °C, using a Lab1st heat exchanger for 18 h. After this time, the reaction was stopped, and the content of the reactor was naturally cooled. Vacuum filtration was used to separate the MOF from the liquid phase. The MOF was placed in a cellulose extraction thimble and placed in a DMF bath at 70 °C for 24 h to remove possible remaining unreacted/excess ligand. The extraction thimble was placed inside a Soxhlet extractor for successive Soxhlet extractions with dichloromethane (DCM, CH_2_Cl_2_, 84.93 g/mol, 99.8%, Sigma-Aldrich, St. Louis, MO, USA) for 24 h. The average extraction cycle was between 60 and 70 min. This procedure allows the DMF to be exchanged with DCM. After the extraction period, the MOF was removed from the cellulose extraction thimble and dried at 120 °C for 18 h inside a Hobersal HD 230 muffle furnace (Forns Hobersal S. L., Barcelona, Spain).

UiO-66-NH_2_ was characterized by Fourier-transform infrared spectroscopy (FTIR), powder X-ray diffraction (PXRD), nitrogen adsorption–desorption at −196 °C, and thermogravimetry analysis (TGA). To obtain the infrared spectrum of UiO-66-NH_2_, a FTIR spectrometer (Spectrum Two, PerkinElmer, Springfield, IL, USA) equipped with an Attenuated Total Reflectance (ATR) accessory was used (16 scans, spectral resolution of 4 cm^−1^). The PXRD analysis of UiO-66-NH_2_ was performed using a diffractometer (D8 Advance, Bruker, Billerica, MA, USA) equipped with a 1D detector (SSD 160) with a nickel filter. The scanning range used was from 5 to 50° (2θ), with a step size of 0.03° and a counting time of 0.5 s. The measurement of nitrogen adsorption–desorption equilibrium at −196 °C was conducted using a gas adsorption analyzer (Quantachrome Instruments, version 10.0, Boynton Beach, FL, USA), which allowed for the determination of different textural properties. The analysis was performed with an activation time of 4 h and an activation temperature of 150 °C. To obtain the TG curve, a thermogravimetric analyzer (TGA 4000, Perkin Elmer) was used, in which the sample was placed in a small alumina pan. During the analysis, the sample was heated at a rate of 2 °C/min, from room temperature up to a maximum of 800 °C, under an air flow of 20 mL/min.

### 2.4. Synthesis of CA22/SiO_2_/UiO-66-NH_2_ Membranes

A CA22/SiO_2_ and four CA22/SiO_2_/UiO-66-NH_2_ membranes were prepared using the phase inversion technique coupled with the sol–gel method [18,19]. The composition of the casting solutions for the membrane synthesis is shown in Table 1. The two digits following CA denote the approximate mass percentage of formamide. After the designation CA22/SiO_2_/UiO-66-NH_2_, the incorporated MOF percentage is indicated. The casting solutions were prepared in glass Schott bottles using cellulose acetate (CA, C_6_H_7_O_2_(OH)_3_, ~30,000 g/mol, ≥97%, Sigma-Aldrich), formamide (CH_3_NO, 45.02 g/mol, Panreac, Barcelona, Spain), pure acetone (C_3_H_6_O, 58.08 g/mol, ≥99.7%, JMGS, LDA, Odivelas, Portugal), TEOS (Si(OC_2_H_5_)_4_, 208.33 g/mol, ≥98%, Alfa Aesar), nitric acid (HNO_3_, 63.01 g/mol, 65% *v*/*v*, Chem-Lab, Zedelgem, Belgium), and UiO-66-NH_2_. The sol–gel silica precursor (TEOS) was added in acidic conditions [18,19].

The UiO-66-NH_2_ MOF was ground and then phased-dispersed in formamide with the help of a vortex before being added to the Schott bottle, where the respective casting solution was prepared. All casting solutions were homogenized for 24 h at 700 rpm at room temperature in a shaker (S50, CAT-Ing, Baden-Württemberg, Germany). The MOFs were further dispersed in the solution through manual stirring and the aid of ultrasound. Since a MOF concentration of 2% already showed evident difficulties in dispersing the MOF in the casting solution, it was decided not to exceed this MOF percentage.

The membrane casting was performed using the phase inversion technique, with the casting solution spread at a constant speed over a glass plate using a 250 μm casting knife. The solvent evaporation time was 30 s, and then the glass plates with the membranes were immersed in a coagulation bath of water at a temperature of 0–5 °C for 2 h. The membranes were stored in a container containing a 15% ethanol solution (*v*/*v*).

### 2.5. Membrane Characterization

**Figure 1:**
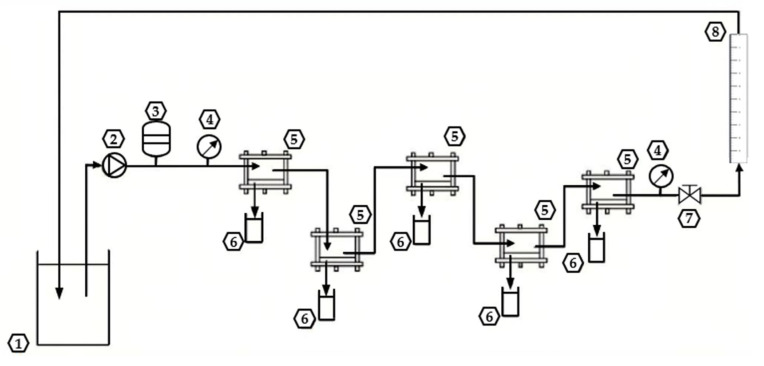
Crossflow Ultrafiltration Installation [20]. Legend: (1) feed tank reservoir, (2) crossflow pump, (3) pressure dumper, (4) manometers, (5) permeation cells, (6) vials, (7) back-pressure valve, (8) rotameter.

CA22/SiO_2_, the CA22/SiO_2_/UiO-66 membrane series and the CA22/SiO_2_/UiO-66-NH_2_ membrane series were characterized in terms of hydraulic permeability, $L_{p}$, molecular weight cut-off (MWCO), and apparent rejection coefficient, f, to pCS and BSA. The permeation experiments were carried out in an ultrafiltration installation depicted in Figure 1, using flat-sheet permeation cells with a membrane surface area of 13.2 cm^2^.

The $L_{P}$ at 25 °C quantifies the permeation capacity of pure water in terms of mass, per unit time, membrane surface area and TMP (transmembrane pressure). The $L_{p}$ is given by Equation (1).

(1) $$L_{P}=\frac{J_{w}}{TMP}$$

where $J_{w}$ is the permeate flux of pure water in kg/(h m^2^) and TMP is the applied transmembrane pressure in bar. The hydraulic permeation experiments were carried out with a volumetric feed flowrate of 2.0 L/min at a TMP ranging from 0.5 to 4 bar.

The membrane molecular weight cut-off (MWCO) was determined through the results obtained in the ultrafiltration of polyethylene glycol (PEG) 1000 (1000 g/mol, Merck, Darmstadt, Germany), PEG 3000 (3000 g/mol, Merck), PEG 6000 (6000 g/mol, Merck), PEG 10,000 (10,000 g/mol, Merck), PEG 20,000 (20,000 g/mol, Merck) and PEG 35,000 (35,000 g/mol, Merck). Each permeation experiment was carried out with aqueous solutions with a concentration of 600 ppm for each PEG under total recirculation mode at the maximum volumetric feed flowrate, 3.5 L/min, a TMP of 1 bar and after 20 min of stabilization. The concentration of solute in both permeate and feed samples was determined using a Total Organic Carbon Analyzer.

The apparent rejection coefficient to solute A is defined by Equation (2), where $C_{AP}$ corresponds to the concentration of solute in the permeate and $C_{AF}$ to the average value between the initial and final solute feed concentration.

(2) $$f_{A}=\frac{C_{AF}-C_{AP}}{C_{AF}}$$

Regarding the rejection of pCS and BSA, distinct permeation tests were conducted: the first test involved 40 ppm pCS solution (molecular weight of 188.2 g/mol), and the second test involved 700 ppm BSA solution (molecular weight of 66,500 g/mol, Sigma-Aldrich). These solutions were prepared in PBS (phosphate-buffered solution). The operating conditions in the permeation tests were a feed circulation flow rate of 3.5 L/min and a TMP of 0.5 bar.

### 2.6. Ultrafiltration of Mixtures of pCS and BSA

In these experiments, 2 L feed solutions were prepared and incubated for 24 h in a thermostatic bath at 37 °C [14] before the permeation assays. During the permeation assay, the feed solutions were also maintained at 37 °C.

After each permeation experiment, the membranes were washed with deionized water at room temperature, maximum feed flow rate and lowest transmembrane pressure until 95% of the initial permeate flux was recovered. This ensures the preservation of the structure of the membrane and of the MOF incorporated.

### 2.7. Evaluation of Free and Protein-Bound pCS in Solutions of pCS and BSA

To quantify the free and protein-bound pCS in the UF feed solutions the Vivaspin^®^ 6 concentrators (30 kDa, PES, Sartorius, Göttingen, Germany) were used as follows:

1. A 5 mL sample from the feed solution was placed in the upper chamber of the concentrator and centrifuged at 7500× *g* for 9 min. After centrifugation, the sample volumes in the upper (retentate) and lower (permeate) compartments of the concentrator were measured. To determine the total amount of protein that was able to permeate, the Bradford’s method was also carried out [21].

2. To quantify the bounded pCS to BSA the salting-out method was performed. Briefly, 1 mL of the concentrator’s retentate volume was taken and added to the required mass of ammonium sulfate ((NH_4_)_2_SO_4_, 132.14 g/mol, Merck, Darmstadt, Germany), to achieve a final concentration of 4.0 M [22]. The resulting solution was centrifuged at 19,000× *g* for 30 min, and the obtained supernatant analyzed for its pCS content.

3. The free pCS was quantified through the analysis of the concentrator’s permeate solution.

## 3. Results and Discussion

### 3.1. UiO-66-NH_2_ Characterization

#### 3.1.1. ATR-FTIR

**Figure 2:**
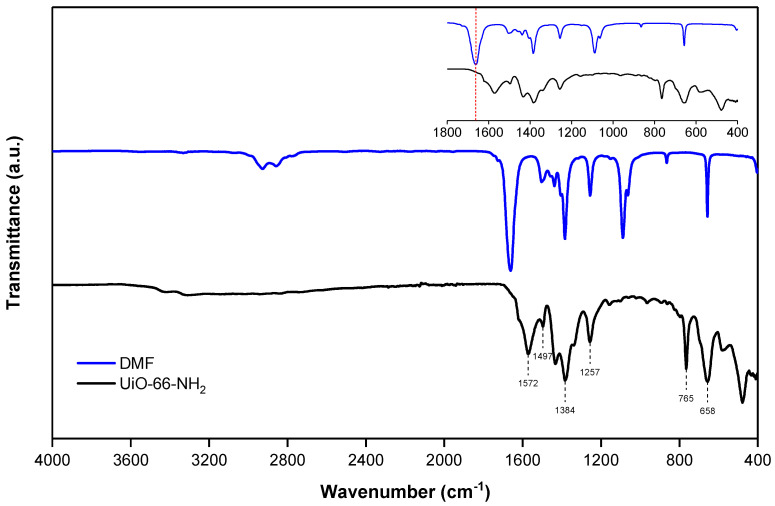
ATR-FTIR spectrum of DMF and the synthesized UiO-66-NH_2_.

The ATR-FTIR spectrum of synthesized UiO-66-NH_2_ is shown in Figure 2. A characteristic band at around 1572 cm^−1^, also observed in UiO-66 [20], was attributed to the asymmetric stretching vibrations of coordinated carboxylate groups, confirming the successful coordination of Zr^4+^ ions with the carboxylic groups of the terephthalate linker [23,24]. The bands at 1497 cm^−1^ and 1384 cm^−1^ were assigned to C=C of the benzene ring and the symmetric O-C-O stretching vibrations of the carboxylate groups, respectively, in agreement with previously reported spectra for UiO-66-NH_2_ [23,24]. The presence of two characteristic bands at 1257 cm^−1^ and 765 cm^−1^ was associated with N-H stretching and bending vibrations, respectively, confirming the successful incorporation of the amino functional group in the framework [23,25]. The band observed at 658 cm^−1^ was attributed to C-H vibrations of the H_2_BDC-NH_2_ linker [23,24,25]. The absence of a band at 1664 cm^−1^ (see red line in the insert of Figure 2), which would correspond to the stretching vibration of C=O bonds from DMF (used as solvent in the synthesis), indicated that solvent removal after extraction and drying was effective [26]. Overall, the FTIR spectrum further supported the successful synthesis of UiO-66-NH_2_.

#### 3.1.2. PXRD

**Figure 3:**
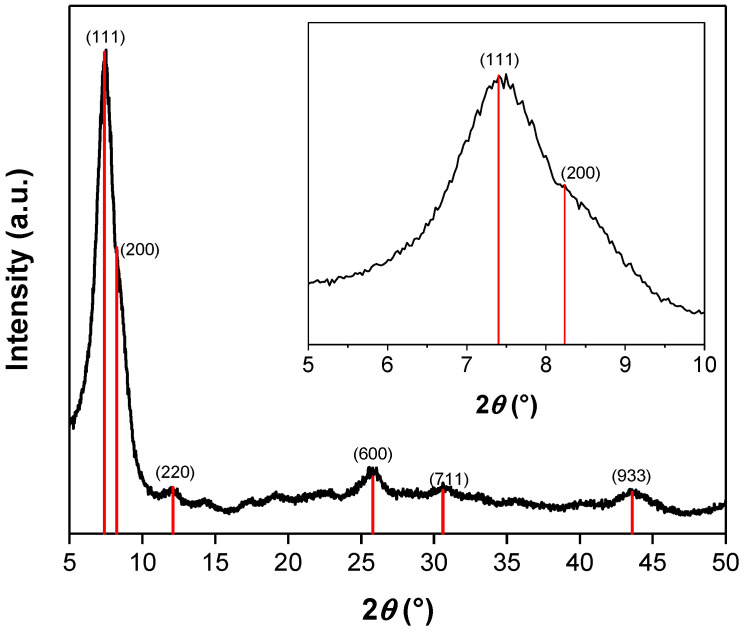
Diffractogram of synthesized UiO-66-NH_2_.

The diffractogram of the synthesized UiO-66-NH_2_ is shown in Figure 3. The positions of relevant peaks reported in the literature for UiO-66-NH_2_ are displayed in red. Based on the diffractogram, it was possible to confirm the crystalline nature of the synthesized MOF. Well-defined peaks were observed at the following 2*θ*: 7.4°, 8.3º, 12.1º, 25.8º, 30.6º, and 43.6º. The peaks observed at these 2*θ* values can be assigned to the (111), (200), (220), (600), (711), and (933) crystallographic planes of UiO-66-NH_2_, respectively, in agreement with previously reported diffraction patterns [23].

Some experimental peaks reported in the literature overlapped with adjacent peaks (see insert of Figure 3), which can be attributed to the presence of broad peaks in the diffractogram. The observation of broad peaks can be associated with the small size of the crystalline domains, which may result from the synthesis protocol employed to obtain particles that can be more homogeneously dispersed in the polymeric matrix. The presence of broad peaks may also help to explain the high external surface area of the material, discussed in the next Section 3.1.3.

#### 3.1.3. Nitrogen Adsorption–Desorption Equilibrium at −196 °C

**Figure 4:**
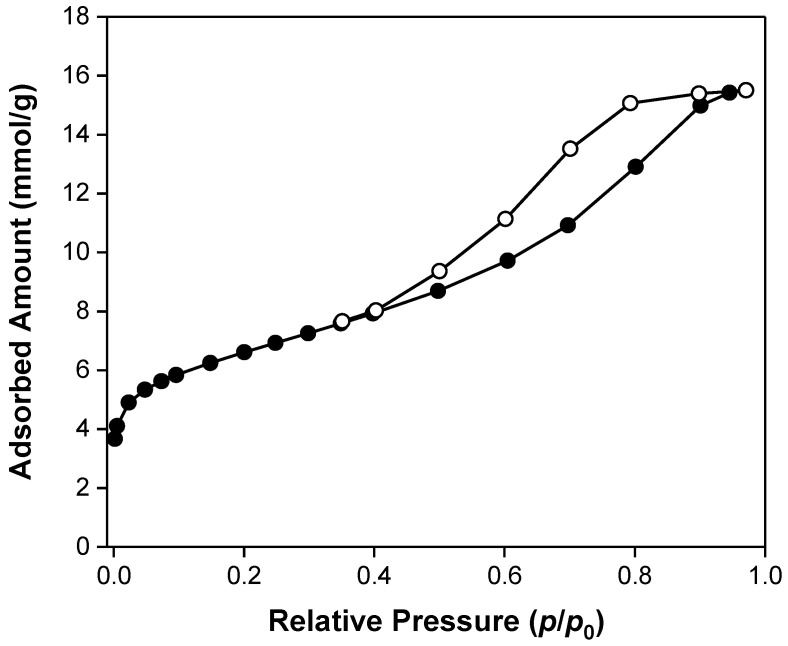
Nitrogen adsorption–desorption equilibrium isotherm at −196 °C for the synthesized UiO-66-NH_2_.

The nitrogen adsorption–desorption isotherm at −196 °C of the synthesized UiO-66-NH_2_ is shown in Figure 4. A mixed Type I and II isotherm, according to the IUPAC classification, was obtained, indicating a predominantly microporous structure associated with a significant external surface area [27]. The presence of a broad hysteresis loop at relative pressures above 0.4 is also another indication of the significant adsorption in the external surface of the particles. This can be associated with interparticle adsorption rather than mesoporosity. This behavior is consistent with the PXRD observations and analysis presented in Section 3.1.2, which indicated the presence of small crystalline domains.

**Figure 5:**
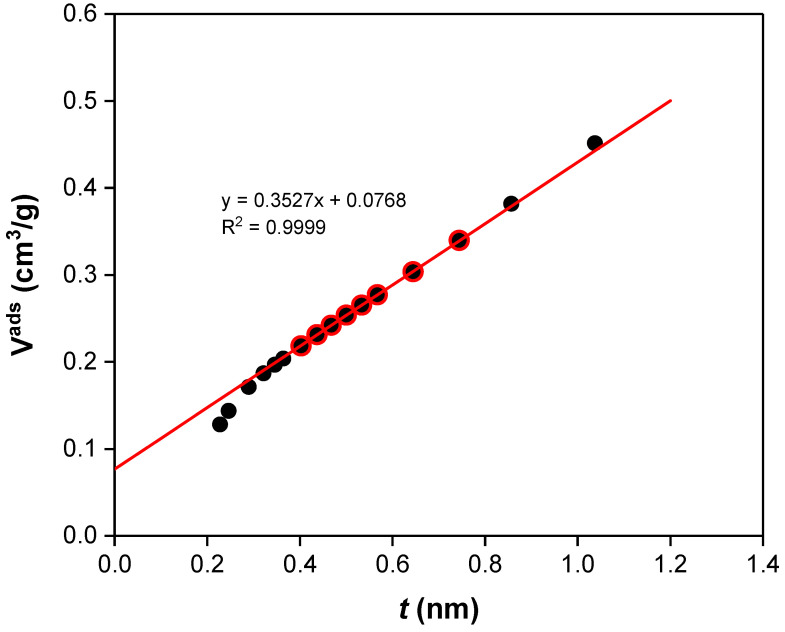
t-plot for nitrogen adsorbed at −196 °C for the synthesized UiO-66-NH_2_.

The BET surface area of the synthesized MOF was 523 m^2^/g. Further analysis of the nitrogen adsorption data at 77 °K using the t-plot method for the synthesized UiO-66-NH_2_, shown in Figure 5, confirmed the high external surface of the sample. The external surface area calculated from this method was 353 m^2^/g, with a microporous volume of 0.077 cm^3^/g. Overall, the nitrogen adsorption data at 77 K indicate that the synthesized UiO-66-NH_2_ is a microporous material with a high external surface area and significant adsorption on interparticle surfaces, which is consistent with the presence of small crystalline domains inferred from the PXRD analysis.

#### 3.1.4. TGA

**Figure 6:**
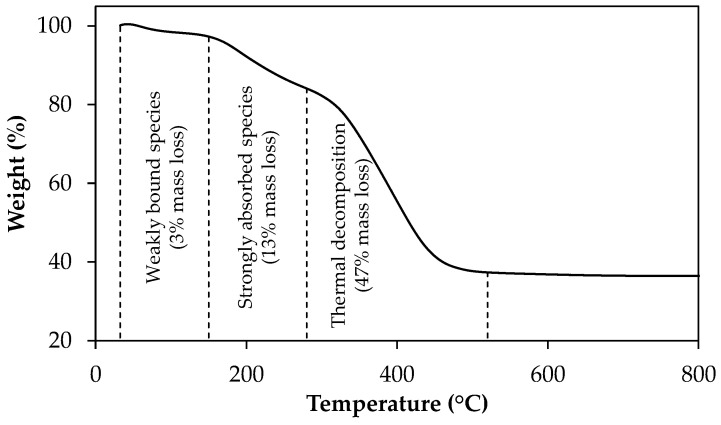
TGA curve of synthesized UiO-66-NH_2_.

The TGA curve of synthesized UiO-66-NH_2_ is shown in Figure 6. A small mass loss of approximately 3% was observed until 150 °C, which was attributed to the removal of weakly bound species, such as residual solvent molecules or weakly adsorbed species. Between 150 and 280 °C, a relatively small mass loss of approximately 13% was observed, which was attributed to the removal of strongly adsorbed species (like intercrystallite water) [28,29]. Considering the boiling point of DMF (153 °C), this result suggests that DMF, if present, exists only in very small quantities, which is consistent with the absence of characteristic DMF bands in the FTIR spectrum and confirms the effective solvent removal after extraction and drying. A significant mass loss of almost 47% occurred between 280 °C and 520 °C, corresponding to the thermal decomposition of the H_2_BDC-NH_2_ organic linkers. At 800 °C, UiO-66-NH_2_ exhibited a remaining total mass of approximately 36%, corresponding to zirconium oxide residue after complete thermal decomposition. This value is lower than the theoretical 42% expected for a defect-free structure. This deviation is consistent with the presence of missing-cluster defects, which can generate additional void space and induce a certain degree of mesoporosity in the MOF.

As shown by the collected TGA data, at 37 °C, a temperature considered relevant to the final application of these membranes, the incorporated MOFs remain unchanged and are thermally stable.

### 3.2. Characterization of CA22/SiO_2_, CA22/SiO_2_/UiO-66, and CA22/SiO_2_/UiO-66-NH_2_ Membranes

#### 3.2.1. Hydraulic Permeability, L_p_, and Molecular Weight Cut-Off, MWCO

The $L_{p}$ and MWCO of CA22/SiO_2_, CA22/SiO_2_/UiO-66 series and CA22/SiO_2_/UiO-66-NH_2_ series membranes are shown in Table 2 and Table 3. Based on the results presented in Table 2, it is observed that, except for the membrane with 1% of UiO-66, there is an increase in hydraulic permeability with the addition of UiO-66. The MWCO shows a gradual increase with the percentage of UiO-66 in the membranes.

Table 3 shows that the membranes incorporating UiO-66-NH_2_ exhibit lower hydraulic permeabilities compared to the CA22/SiO_2_ membrane and to the CA22/SiO_2_/UiO-66 series of membranes. The MWCO values are also lower. Although the UiO-66-NH_2_ MOF is more hydrophilic than the UiO-66 [30], its incorporation in membranes reduces both hydraulic permeability and MWCO. An explanation may be the different ratio of the average membrane pore size to the MOF size.

#### 3.2.2. Ultrafiltration of Single Solute Solutions

The rejection coefficients to pCS and to BSA are presented in Table 4 for the membranes CA22/SiO_2_ and the CA22/SiO_2_/UiO-66 series and in Table 5 for CA22/SiO_2_ membrane and the series of CA22/SiO_2_/UiO-66-NH_2_ membranes.

From the analysis of Table 4 and Table 5, it is evident that the incorporation of UiO-66 or UiO-66-NH_2_ in the membranes results in lower rejection coefficients to pCS and therefore leads to the enhancement of its removal capacity. Increasing the percentage of MOF incorporation decreases rejection coefficients to pCS. Regarding the membrane rejection coefficients to BSA, the results show that BSA is almost completely rejected.

### 3.3. Ultrafiltration of Solutions with Two Solutes, pCS and BSA

The composition of a solution with the PBUT, pCS, and BSA is chosen on the basis of the average concentration of pCS found in patients with CKD, which is 568 µM, and BSA concentration of 526.9 μM, which is the average concentration of Human Serum Albumin (HSA) in blood plasma.

#### 3.3.1. Quantification of Free and BSA Bounded pCS in the UF Feed Solutions

**Figure 7:**
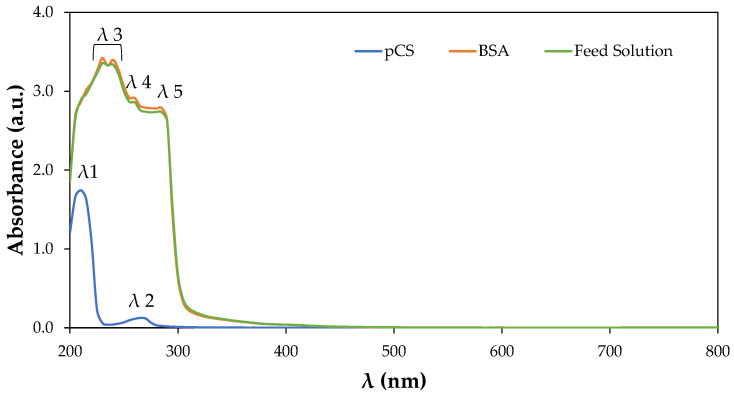
Absorbance spectra of the standard solutions of 568 µM pCS and 526.9 µM BSA in PBS, and of the UF feed solution (100 ppm pCS and 35 g/L BSA). λ1: 210 nm, λ2: 265 nm, λ3: 230–240 nm, λ4: 260 nm, λ5: 280 nm.

**Figure 8:**
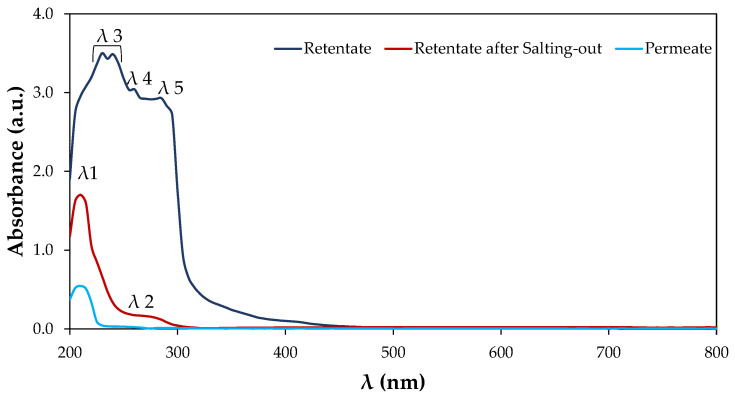
Absorbance spectra of the permeate and retentate obtained after centrifugation in the Vivaspin^®^ 6 concentrator, and of the retentate after application of the salting-out method (samples of steps 2 and 3 of the Section 2.7 protocol); λ1: 210 nm, λ2: 265 nm, λ3: 230–240 nm, λ4: 260 nm, λ5: 280 nm.

Following the procedure described in point 2.7., the first step of the feed sample centrifugation in the 30 kDa membrane of the Vivaspin^®^ 6 yields a retentate of 3 mL and a permeate of 2 mL. The concentration of pCS in the retentate and permeate was 860 ± 10 μM and 132 ± 2 μM, respectively. The absorbance spectra of the standard solutions of BSA, pCS and bounded pCs (UF feed solution) in PBS, as well as of the permeate and retentate before and after the salting-out method are shown accordingly in Figure 7 and Figure 8. In the pCS spectrum, the absorption peaks λ1 and λ2 are characteristic of the π→π* transitions in phenolic compounds [31]. Peak λ2 was chosen for pCS quantification in the permeate and retentate after salting-out. The characteristic absorption peak in any protein spectrum is λ5, which corresponds to absorption of the aromatic side chains of the protein aminoacids (Trp, Tyr, and Phe), and is commonly used to determine protein concentrations. Together with λ3, conformational changes to the proteins, including bonding to other molecules such as pCS, can also be monitored [32,33]. Peak λ4 may come from the vestigial residues of DNA from mesophilic germs present in the original container [34].

The absorption spectrum of the UF feed solutions is similar to the BSA standard. The absorption peaks of the pCS present in solution are fully hidden by the stronger absorption bands of BSA making its direct identification and quantification impossible by spectroscopy, as shown in Figure 7.

To determine the pCS concentration in the retentate, the salting-out method was carried out to cause BSA precipitation (step 2 of Section 2.7). The pCS concentration which was bonded to BSA was 515 ± 6 μM. The free pCS which permeated through the Vivaspin^®^ 6 membrane was also quantified, being its final concentration 51 ± 1 μM (step 3 of Section 2.7). The presence of 3.0% BSA peptides was also detected in the permeate by the Bradford’s method; however, as shown in Figure 8, these peptides remained unbound from the pCS since the permeate absorption profile is similar to the pCS standard solution. The sum of the free and bound pCS was 566 ± 6 μM, which is in accordance with the concentration value of 568 μM of the prepared UF feed sample, with a very low deviation of 0.4%.

#### 3.3.2. Ultrafiltration of Two Solutes, pCS and BSA, Solutions with CA22/SiO_2_, CA22/SiO_2_/UiO-66, and CA22/SiO_2_/UiO-66-NH_2_ Membranes

As stated in Section 3.3, the pCS and BSA concentrations in the UF feed solution were chosen to simulate a CKD patient situation. The rejection coefficients, $f_{pCS}$, to pCS and the percentage of removal of the pCS bound to BSA, $\%{pCS}_{bound\;removed},$ were determined by Equations (3) and (4), respectively.

(3) $$f_{pCS}=\frac{{\left[pCS\right]\;}_{feed\;solution}-{\left[pCS\right]}_{permeate\;}}{{\left[pCS\right]\;}_{feed\;solution}}$$

(4) $$\%{pCS}_{bound\;removed}=\frac{{\left[pCS\right]\;}_{collected\;in\;the\;permeate\;}-{\left[pCS\right]}_{free\;in\;feed\;}}{{\left[pCS\right]}_{bound\;in\;feed}}\times 100\;$$

**Figure 9:**
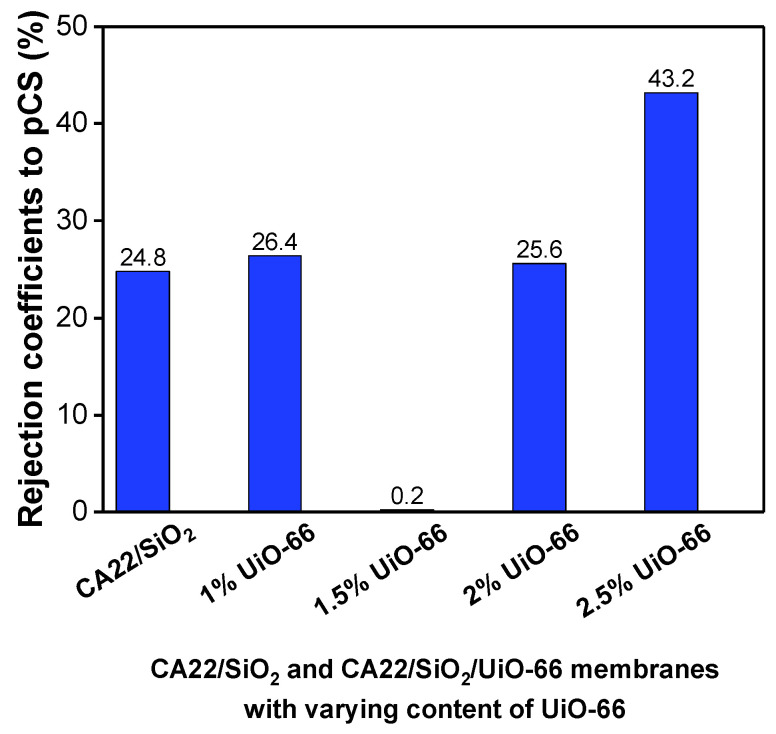
Rejection coefficients to pCS, $f_{pCS}$, in percentage, for the ultrafiltration of two solutes solution, 568 μM pCS and 526.9 μM BSA, with the CA22/SiO_2_ membrane and the CA22/SiO_2_/UiO-66 membranes with varying content of UiO-66.

**Figure 10:**
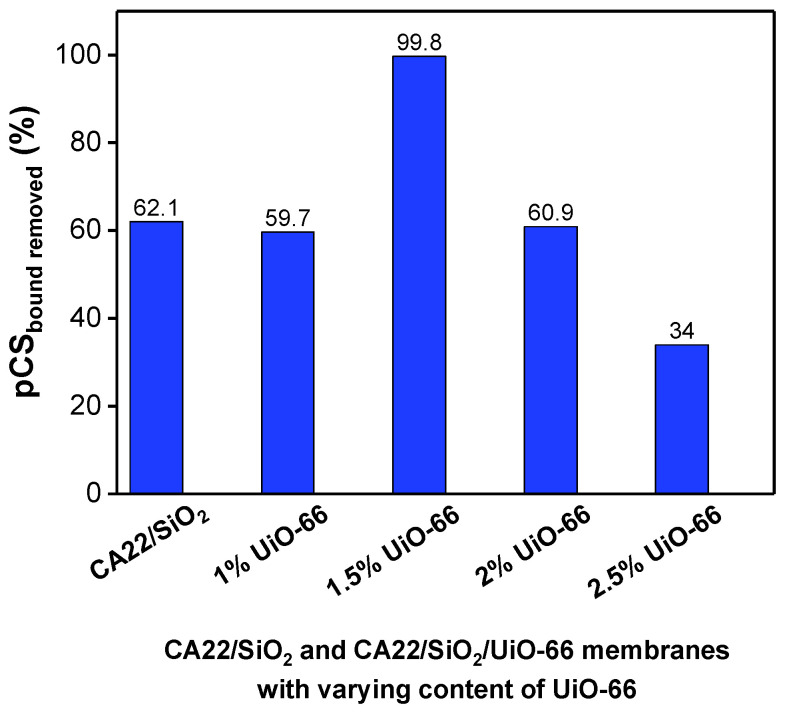
The percentage removal of pCS bound to BSA by the ultrafiltration of a solution of 568 μM pCS and 526.9 μM BSA with the CA22/SiO_2_ membrane and the CA22/SiO_2_/UiO-66 membranes with varying content of UiO-66.

Rejection coefficients to pCS, $f_{pCS}$, in percentage, are shown in Figure 9 for the CA22/SiO_2_ membrane and for the CA22/SiO_2_/UiO-66 membranes with varying content of UiO-66. The removal percentage of the pCS bound to BSA, $\%{pCS}_{bound\;removed},$ is shown in Figure 10 for the CA22/SiO_2_ membrane and for the CA22/SiO_2_/UiO-66 membranes with varying content of UiO-66.

In Figure 9, the CA22/SiO_2_/UiO-66 1.5% membrane stands out with rejection coefficient to pCS close to zero (0.2%) and, therefore, nearly removing 100% pCS. This is in accordance with the result in Figure 10 where the CA22/SiO_2_/UiO-66 1.5% membrane displays a capacity of removing 99.8% of the pCS bound to BSA.

**Figure 11:**
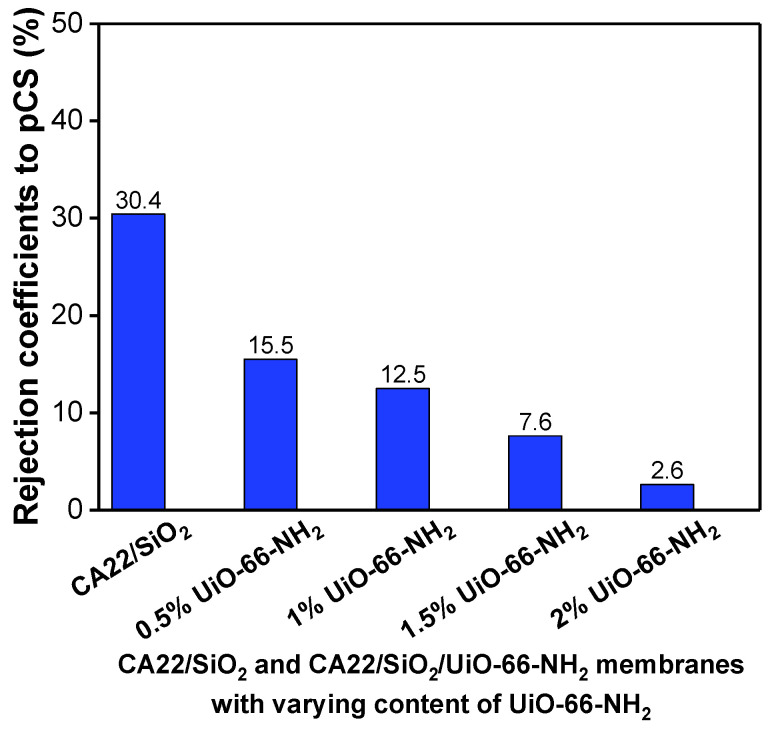
Rejection coefficients to pCS, $f_{pCS}$, in percentage, for the ultrafiltration of two solutes solution, 568 μM pCS and 526.9 μM BSA with the CA22/SiO_2_ membrane and the CA22/SiO_2_/UiO-66-NH_2_ membranes with varying content of UiO-66-NH_2_.

**Figure 12:**
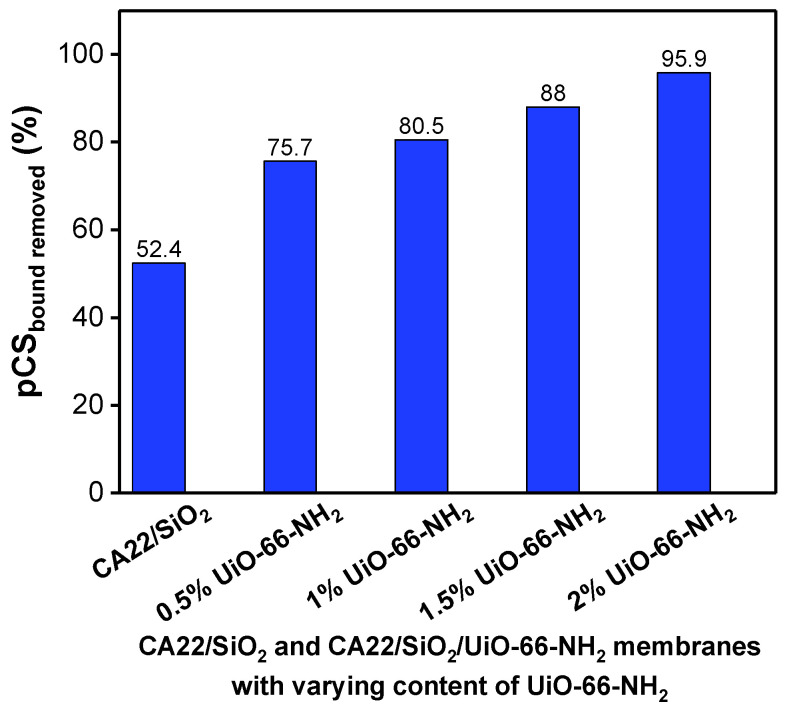
The percentage removal of pCS bound to BSA by the ultrafiltration of a solution with 568 μM pCS and 526.9 μM BSA with the CA22/SiO_2_ membrane and the CA22/SiO_2_/UiO-66-NH_2_ membranes with varying content of UiO-66-NH_2_.

Considering the mixed matrix membranes incorporating UiO-66-NH_2_, Figure 11 displays the rejection coefficients to pCS, $f_{pCS}$, in percentage, for the CA22/SiO_2_ membrane and for the CA22/SiO_2_/UiO-66-NH_2_ membranes with varying content of UiO-66-NH_2_. The removal percentage of the pCS bound to BSA, $\%{pCS}_{bound\;removed},$ is shown in Figure 12 for the CA22/SiO_2_ membrane and for the CA22/SiO_2_/UiO-66-NH_2_ membranes with varying content of UiO-66-NH_2_.

Figure 11 shows that the rejection to pCS by membranes with UiO-66-NH_2_ decreases as the percentage of MOF increases, reaching 2.6% for the CA22/SiO_2_/UiO-66-NH_2_ 2% membrane. Thus, the CA22/SiO_2_/UiO-66-NH_2_ 2% membrane, in a scenario simulating a hemodialysis session in a patient with CKD, is the most effective in removing 95.9% of pCS bound to BSA, as shown in Figure 12.

When comparing the performance of the CA22/SiO_2_/UiO-66 and CA22/SiO_2_/UiO-66-NH_2_ membrane series, it is notable that the CA22/SiO_2_/UiO-66-NH_2_ 1% and CA22/SiO_2_/UiO-66-NH_2_ 2% membranes show lower pCS rejection coefficients than the CA22/SiO_2_/UiO-66 1% and CA22/SiO_2_/UiO-66 2% membranes, respectively.

In terms of the removal capacity of pCS bound to BSA, the CA22/SiO_2_/UiO-66-NH_2_ 2% membrane and the CA22/SiO_2_/UiO-66 1.5% membrane display the highest values and makes them promising candidates for the removal of the PBUT, pCS, bound to BSA in hemodialysis.

## 4. Conclusions

The synthesis of ultrafiltration mixed matrix membranes is achieved through the incorporation of MOFs in a hemocompatible matrix of cellulose acetate/silica. The content of the MOF UiO-66 ranges from 1% to 2.5% and of the MOF UiO-66-NH_2_ from 0.5% to 2.0%.

The extensive characterization carried out in both membrane types show that the addition of UiO-66 enhanced the hydraulic permeability, and the molecular weight cut-off in comparison with the ones incorporating UiO-66-NH_2_.

The membranes with 1.5% UiO-66 and 2% UiO-66-NH2 show a rejection coefficient close to 100% removal of pCS, 0.2% and 2.6%, respectively.

The removal capacity of pCS bound to BSA is 99.8% for the membrane with 1.5% of UiO-66 and 95.9% for the membrane with 2.0% of UiO-66-NH_2_.

The high removal capacity demonstrated by these mixed matrix membranes for the extraction of the PBUT, pCS, is 15% higher than the values reported in the literature [35]. These membranes are a strong asset for the pursuit of further studies with other PBUTs, and their future application in hemodialysis-based treatments.

## Figures and Tables

**Table 1:** Composition (wt.%) of the casting solutions for the synthesis of CA22/SiO_2_ and CA22/SiO_2_/UiO-66-NH_2_, with MOF loadings from 0.5 to 2%.

Membranes	CA22/SiO_2_	CA22/SiO_2_/UiO-66-NH_2_ 0.5%	CA22/SiO_2_/UiO-66-NH_2_ 1%	CA22/SiO_2_/UiO-66-NH_2_ 1.5%	CA22/SiO_2_/UiO-66-NH_2_ 2%
CA	16.4	16.3	16.2	16.2	16.1
Formamide	21.3	21.1	21.0	20.9	20.7
Acetone	58.8	58.6	58.3	57.9	57.7
UiO-66-NH_2_	-	0.5	1.0	1.5	2
TEOS	3	3	3	3	3
Water	0.5	0.5	0.5	0.5	0.5
HNO_3_	3 drops (pH ~ 2)	3 drops (pH ~ 2)	3 drops (pH ~ 2)	3 drops (pH ~ 2)	3 drops (pH ~ 2)

**Table 2:** Characterization parameters of the CA22/SiO_2_ membrane and the CA22/SiO_2_/UiO-66 membranes with varying content of UiO-66.

Parameter	CA22/SiO_2_	CA22/SiO_2_/UiO-66 1%	CA22/SiO_2_/UiO-66 1.5%	CA22/SiO_2_/UiO-66 2%	CA22/SiO_2_/UiO-66 2.5%
$L_{p}\;(kgm^{-2}h^{-1}bar^{-1})$	15.0	12.1	34.3	19.8	29.8
MWCO (kDa)	4.68	7.31	9.50	15.0	16.4

**Table 3:** Characterization parameters of the CA22/SiO_2_ membrane and the CA22/SiO_2_/UiO-66-NH_2_ membranes with varying content of UiO-66-NH_2_.

Parameter	CA22/SiO_2_	CA22/SiO_2_/UiO-66-NH_2_ 0.5%	CA22/SiO_2_/UiO-66-NH_2_ 1%	CA22/SiO_2_/UiO-66-NH_2_ 1.5%	CA22/SiO_2_/UiO-66-NH_2_ 2%
$L_{p}\;(kgm^{-2}h^{-1}bar^{-1})$	16.8	5.11	4.07	8.20	4.07
MWCO (kDa)	5.88	4.59	4.19	4.53	4.65

**Table 4:** Rejection coefficients to pCS and to BSA for CA22/SiO_2_ and CA22/SiO_2_/UiO-66 membranes with different UiO-66 content.

Membrane	*f*_pCS_ (%)	*f*_BSA_ (%)
CA22/SiO_2_	15.7	98.1
CA22/SiO_2_/UiO-66 1%	5.3	98.5
CA22/SiO_2_/UiO-66 1.5%	1.2	98.1
CA22/SiO_2_/UiO-66 2%	0.5	98.1
CA22/SiO_2_/UiO-66 2.5%	0.07	98.2

**Table 5:** Rejection coefficients to pCS and to BSA for CA22/SiO_2_ and CA22/SiO_2_/UiO-66-NH_2_ membranes with different UiO-66-NH_2_ content.

Membrane	*f*_pCS_ (%)	*f*_BSA_ (%)
CA22/SiO_2_	15.1	98.2
CA22/SiO_2_/UiO-66-NH_2_ 0.5%	8.3	99.1
CA22/SiO_2_/UiO-66-NH_2_ 1%	4.1	99.1
CA22/SiO_2_/UiO-66-NH_2_ 1.5%	2.0	99.2
CA22/SiO_2_/UiO-66-NH_2_ 2%	0.4	99.2

## Data Availability

The original contributions presented in this study are included in the article. Further inquiries can be directed to the corresponding author.
